# Current Predictive Resting Metabolic Rate Equations Are Not Sufficient to Determine Proper Resting Energy Expenditure in Olympic Young Adult National Team Athletes

**DOI:** 10.3389/fphys.2021.625370

**Published:** 2021-02-04

**Authors:** Aydın Balci, Ebru Arslanoğlu Badem, Ayfer Ezgi Yılmaz, Aslı Devrim-Lanpir, Bihter Akınoğlu, Tuğba Kocahan, Adnan Hasanoğlu, Lee Hill, Thomas Rosemann, Beat Knechtle

**Affiliations:** ^1^Department of Sports Medicine, Ankara Yıldırım Beyazıt University, Yenimahalle Training and Research Hospital, Ankara, Turkey; ^2^Department of Health Services, Sports General Directorship, The Ministry of Youth and Sports, Center of Athlete Training and Health Research, Ankara, Turkey; ^3^Department of Statistics, Hacettepe University, Ankara, Turkey; ^4^Department of Nutrition and Dietetics, Faculty of Health Sciences, Istanbul Medeniyet University, Istanbul, Turkey; ^5^Department of Physiotherapy and Rehabilitation, Faculty of Health Sciences, Ankara Yıldırım Beyazıt University, Ankara, Turkey; ^6^Division of Gastroenterology and Nutrition, Department of Pediatrics, McMaster University, Hamilton, ON, Canada; ^7^Institute of Primary Care, University of Zurich, Zurich, Switzerland

**Keywords:** indirect calorimetry, resting metabolic rate, predictive equations, low energy availability, olympic athletes

## Abstract

Predictive resting metabolic rate (RMR) equations are widely used to determine athletes’ resting energy expenditure (REE). However, it remains unclear whether these predictive RMR equations accurately predict REE in the athletic populations. The purpose of the study was to compare 12 prediction equations (Harris-Benedict, Mifflin, Schofield, Cunningham, Owen, Liu’s, De Lorenzo) with measured RMR in Turkish national team athletes and sedentary controls. A total of 97 participants, 49 athletes (24 females, 25 males), and 48 sedentary (28 females, 20 males), were recruited from Turkey National Olympic Teams at the Ministry of Youth and Sports. RMR was measured using a Fitmate GS (Cosmed, Italy). The results of each 12 prediction formulas were compared with the measured RMR using paired *t*-test. The Bland-Altman plot was performed to determine the mean bias and limits of agreement between measured and predicted RMRs. Stratification according to sex, the measured RMR was greater in athletes compared to controls. The closest equation to the RMR measured by Fitmate GS was the Harris-Benedict equation in male athletes (mean difference -8.9 (SD 257.5) kcal/day), and Liu’s equation [mean difference -16.7 (*SD* 195.0) kcal/day] in female athletes. However, the intra-class coefficient (ICC) results indicated that all equations, including Harris-Benedict for male athletes (ICC = 0.524) and Liu’s for female athletes (ICC = 0.575), had a moderate reliability compared to the measured RMR. In sedentary subjects, the closest equation to the measured RMR is the Nelson equation in males, with the lowest RMSE value of 118 kcal/day [mean difference: 10.1 (*SD* 117.2) kJ/day], whereas, in females, all equations differ significantly from the measured RMR. While Nelson (ICC = 0.790) had good and Owen (ICC = 0.722) and Mifflin (calculated using fat-free mass) (ICC = 0.700) had moderate reliability in males, all predictive equations showed poor reliability in females. The results indicate that the predictive RMR equations failed to accurately predict RMR levels in the participants. Therefore, it may not suitable to use them in determining total energy expenditure.

## Introduction

Resting metabolic rate (RMR) is an essential component of daily energy needs and accounts for approximately 60–70% of total energy expenditure in sedentary individuals (Johnstone et al., 2005). The accurate determination of daily energy needs is vital in maintaining an optimal body composition and developing nutritional strategies for providing body requirements (Thomas et al., 2016). While a positive energy balance increases body weight and causes weight-related health issues such as obesity and metabolic syndrome (Spiegelman and Flier, 2001; Romieu et al., 2017), a negative energy balance may result in several nutrient deficiencies, fatigue, disordered body image, and muscle mass loss (Black et al., 2018).

Providing sufficient energy availability is one of the most critical points for boosting sports performance, physiological function, and maintaining metabolic health (Loucks et al., 2011). Low energy availability (LEA) has been well-defined in recent years by the International Olympic Committee (IOC), emphasizing its impact on athletes’ health (Mountjoy et al., 2015). LEA is the major detrimental factor underpinning several unfavorable health outcomes, including menstrual disorders, reduced bone-mineral density, hormonal dysregulation, and other metabolic disorders specifically in the female athletic population (Mountjoy et al., 2018). RMR is recognized as one of the key determinants in assessing an athlete’s energy needs (Loucks et al., 2011). Accurate RMR measurement is a crucial component in determining optimal energy needs in order to prepare a comprehensive person-specific sports nutrition program (Jagim et al., 2018). Thus, an optimal nutritional strategy developed according to precise energy needs provides pre/post-training needs, decreases fatigue, and up-regulates body compositions while improving athletes’ metabolic functions (Thomas et al., 2016).

It is well-known that indirect calorimetry (IC) is the gold standard to measure RMR by measuring oxygen consumption and carbon dioxide production (Da Rocha et al., 2006). In recent years, several alternative portable indirect calorimeters have been developed aiming to provide a cost-effective, easy, and accurate measurement. However, these calorimeters only measure oxygen consumption and assume respiratory quotient as 0.85. Therefore, while hand-held calorimeters present controversial results (Compher et al., 2005), portable desktop indirect calorimeters such as Fitmate GS provide better results for measuring RMR in the healthy population (Nieman et al., 2006; Vandarakis et al., 2013).

Several factors such as a high equipment cost, the need for trained personnel, and long measurement times limit the use of indirect calorimetry in practice (Compher et al., 2006). Thus, various equations such as Harris-Benedict (Harris and Benedict, 1918), Mifflin (Mifflin et al., 1990), Schofield (ten Haaf and Weijs, 2014), Cunningham (Cunningham, 1991), Owen (Miller et al., 2013), Liu’s (Liu et al., 1995), De Lorenzo (De Lorenzo et al., 1999), Bernstein (Bernstein et al., 1983), Johnstone (Johnstone et al., 2006), Roza (Roza and Shizgal, 1984) and Nelson (Nelson et al., 1992) have been developed to estimate RMR by regression analyses using various variables such as body weight, body height, age, sex, and fat-free mass (FFM). Using these equations to estimate RMR can be a cost-effective and time-saving strategy. Accordingly, the American College of Sports Medicine (ACSM) has suggested that the Harris-Benedict and Cunningham equations were the most appropriate to determine RMR in the athletic population (Thomas et al., 2016). However, these equations have been found to underestimate RMR in some athletic populations, such as the Harris-Benedict equation in ultra-endurance athletes (Devrim-Lanpir et al., 2019) and the Cunningham and Harris-Benedict equations in elite rowers and canoeists (Carlsohn et al., 2011). Studies investigating the validity of predictive RMR equations in groups of several generations and metabolic conditions have revealed that these equations are not valid at the same level in all groups (Delsoglio et al., 2019; Schofield et al., 2019). Therefore, before using a predictive RMR equation in a group, it should be determined whether they are suitable for the group in question.

With the hypothesis that RMR may vary with physical activity level and sex, the present study aimed to estimate RMR using Harris-Benedict, Mifflin, Schofield, Cunningham, Owen, Liu’s, and De Lorenzo equations and to compare the results with the measured RMR (Fitmate GS) in Turkish national team athletes and their sedentary counterparts. Additionally, we aimed to determine whether these equations were suitable for predicting RMR in both groups.

## Materials and Methods

### Participants

A total of 97 participants, 49 Turkish Olympic young adult national team athletes [25 males (19.1 ± 1.5 years; 178.7 ± 6.1 cm; 75.4 ± 12.4 kg; 66.7 ± 7.6 kg of FFM; 10.6 ± 3.9 BF (%)), 24 females (20.3 ± 2.1 years; 163.3 ± 6.6 cm; 60.6 ± 12.7 kg; 47.0 ± 5.7 kg of FFM; 19.9 ± 4.5 BF (%))], and 48 sedentary [28 males (19.9 ± 1.4 years; 176.8 ± 5.5 cm; 78.3 ± 12.8 kg; 62.1 ± 5.7 kg of FFM; 19.6 ± 4.1 BF (%)), 28 females (20.1 ± 1.6 years; 163.4 ± 4.1 cm; 60.0 ± 10.3 kg; 43.4 ± 4.5 kg of FFM; 26.7 ± 6.3 BF (%))], were included in the study. The subjects were recruited from the Turkey National Olympic Teams at the Ministry of Youth and Sports between January 2020 and March 2020. Participants were informed about the study in detail, and verbal and written informed consent of the participants and/or their legal representatives was obtained prior to enrollment. The sample size was calculated by using the following formula developed to calculate the sample size in pilot trials; *n* = ln (1 - γ). ln (1 - π) - 1 (Viechtbauer et al., 2015). At a power of 0.85 with 95% confidence, 19 subjects with each group and sex were necessary [γ = 0.95 (95% confidence level)] and [π = 0.15 (85% power)].

The inclusion criteria of the study for athletes were: (1) participation in Turkish National team sports for at least 1 year, (2) high physical activity level according to Total Physical Activity Score (TPAS), (3) no history of any metabolic disorders (4) no current injuries or ongoing therapies, and (5) aged between 18 and 25 years. Athletes were recruited from several sporting disciplines including track and field (4 men; 4 women), long-distance swimming (4 men, 2 women), modern pentathlon (1 men, 4 women), fencing (1 men, 2 women), karate (5 men, 5 women), taekwondo (5 men), boxing (3 men, 3 women), and soccer (2 men, 4 women). The inclusion criteria for the sedentary subjects were: (1) low physical activity level according to Total Physical Activity Score (TPAS), (2) no history of any metabolic disorders, and (3) no current injuries or ongoing therapies.

The participants were examined by a sports medicine specialist prior to commencing the study. Participants who were determined to have any current health issues or chronic disease history were excluded from the study. The International Physical Activity Questionnaire-Short Form (IPAQ-SF) was applied to the participants to assess their physical activity level (Craig et al., 2003; Saglam et al., 2010). Participants with moderate TPAS scores were also excluded from the study.

### Design

The study was planned as a cross-sectional design of Turkish national and Olympic athletes and matched (sex, age, and BMI) sedentary controls.

### Study Procedure

All procedures of the study were conducted in accordance with the Declaration of Helsinki. The ethics committee approval was obtained from the University Ethics Committee (2020/37).

All participants were required to visit the performance laboratory once between 8:00 a.m. and 9:00 a.m. Both body composition and RMR measurements were performed on the same day. The study was conducted at the Center of Athlete Training and Health Research of the Ministry of Youth and Sports.

#### International Physical Activity Questionnaire-Short Form (IPAQ-SF)

IPAQ-SF was used to determine the physical activity levels of the participants (Craig et al., 2003; Saglam et al., 2010). The questionnaire consists of seven questions in four parts and has been validated for adults aged 18–69 years.

The questionnaire aims to determine when a participant was physically active in the past 7 days. More specifically, questions were pertaining to the frequency and duration of each physical activity level (sitting, walking, moderate- or high-intensity) performed in the last 7 days. The physical activity level is determined by the MET (3.5 ml oxygen consumption per kg per minute at rest = 1 MET = 3.5 ml/kg/min) method. The Total Physical Activity Score (TPAS) of the participants were calculated using the following equations:

• Walking Score (MET-min/week) = 3.3 × duration (min) × frequency (day)
• Moderate-Intensity Physical Activity Score (MET-min/week) = 4.0 × duration (min) × frequency (day)
• High-Intensity Physical Activity Score (MET-min/week) = 8.0 × duration (min) × frequency (day)
• TPAS (MET- MET-min/week) = Walking Score + Moderate-Intensity Physical Activity Score + High-Intensity Physical Activity Score

Participants were then divided into three groups according to their TPAS as low, moderate, and high physical activity levels. According to these groups;

• Low: TPAS < 600 MET-min/week
• Moderate: 600 MET-min/week < TPAS < 3,000 MET-min/week
• High: TPAS > 3,000 MET-min/week.

#### Body Composition Measurement

Body composition, including body weight (kg), BMI (kg/m^2^), body fat percentage (%), and fat-free mass (FFM) (kg/m^2^) were measured using the Bioelectrical Impedance Analysis (BIA) (Tanita MC-980, 1,000 kHz, 0.1 accuracy, Japan). Participants were asked to visit the laboratory in a fasted state (at least 4 h), have refrained from caffeine (at least 4 h), alcohol (at least 2 h), and cigarettes (at least 2 h). Further, participants were required to not exercise at a high intensity for at least 24 h prior to the measurements (Compher et al., 2006). Additionally, to ensure that all subjects were in an euhydrated state, we asked the sedentary subjects to drink 3.7 L of water a day for males and 2.7 L a day for females before the test day (Institute of Medicine [US], 2005). We informed the Olympic athletes about continuing their individual hydration strategies (Kenefick, 2018). On the morning of the test, we checked the urine specific gravity of all subjects using a semi-automatic reflectance photometry (Mission 500 Urine analyzer, United States), and the color of the urine using a urine color scale. All tests were performed after ensuring that all subjects were in a euhydrated state. All participants except three male athletes and two sedentary women presented adequate hydration status before the measurement. For these three dehydrated athletes, we checked the fluid intake strategies, exercise intensity, and water and fluid consumption throughout the day before the measurement. We made certain suggestions for regulating the hydration states, then repeated the measurements 3 days after the first measurement day. For dehydrated sedentary women, we found that they did not consume the recommended water and fluid intake. We stated them to consume 2.7 L of water a day. Measurements were repeated 3 days after the first measurement day.

#### RMR Measurement

RMR was measured using Fitmate GS (Cosmed, Rome, Italy). We set the environmental characteristics before each measurement in line with the remarkable review by Compher et al. (2006). All RMR measurements were performed in a dimly lit, quiet room with controlled room temperature (22.3 ± 0.9°C) and relative humidity (40.7 ± 1.2%). The oxygen sensor of the Fitmate GS metabolic cart was tested by manufacturer representatives using calibration gases (room air and reference O_2_ gas) before the measurement period in order to verify an optimal machine functioning. Both calibration gases were run through the metabolic system to check for the drift of the O2 analyzer. The Fitmate GS measured the room air and reference O_2_ gases at 20.91% (actual O_2_ percentage of the room air: 20.93%) and 16.03% (the O_2_ percentage of the reference O_2_ gas: 16.0%), respectively, revealing that the Fitmate GS metabolic cart was valid and accurate to use in the study. We performed a maximum of two RMR measurements per day and run manually a flowmeter calibration once per week according to the manufacturer recommendation. The Fitmate GS metabolic cart also automatically self-calibrated (up to 5 min) before each test.

During the test procedure, subjects were asked to lie in a supine position on a stretcher to rest without falling asleep for 20 min, in a silent, dusk room with an ambient temperature of 20–25°C. The researchers performed a flowmeter calibration before each measurement. The canopy hood (headgear) of the Fitmate GS device was wearied to the participants. The measurement lasted for 30 min to achieve a steady-state (the state that Coefficient of Variation (CV) in VO_2_ is <10% during the 30 min measurement [discarding the first 5 min)]. The Fitmate GS metabolic monitor device does not contain a carbon dioxide sensor. Therefore, it calculates the RMR by estimating CO_2_ production from a fixed RQ of 0.85 based on the abbreviated Weir equation (Weir, 1949).

#### Calculation of Resting Metabolic Rate With Prediction Equations

The measured RMR was compared to the predictive RMR calculated by widely used predictive equations, including weight-based [Harris-Benedict (Harris and Benedict, 1918) (age, weight, height), Mifflin (Mifflin et al., 1990) (age, weight, height), Schofield (ten Haaf and Weijs, 2014) (weight), Owen (Miller et al., 2013) (weight), Liu’s (Liu et al., 1995) (age, weight, height), and De Lorenzo (De Lorenzo et al., 1999) (weight and height)] and Roza (Roza and Shizgal, 1984) (age, weight, height) equations, and FFM-based equations [Cunningham (Cunningham, 1991) (FFM), Mifflin (Mifflin et al., 1990) (FFM), Bernstein (Bernstein et al., 1983) (FFM, FM, age), Nelson (Nelson et al., 1992) (FFM, FM), and Johnstone (Johnstone et al., 2006) (FFM, FM, age)].

### Statistical Analysis

All the study data were presented as mean ± *SD*. The Shapiro-Wilks test was used to determine the normal distribution of data, and the Levene test was used to investigate the homogeneity of the variances. Data were analyzed separately according to sex and physical activity level. Differences between the groups were investigated with the Independent *t*-test. The Kruskal-Wallis test was applied when the assumption of normality was not provided. A paired sample *t*-test was applied to compare the measured RMR and the results of the 12 prediction equations one by one. The Bland Altman plot was performed to determine mean bias and limits of agreement between measured and predicted RMRs. The intra-class correlation coefficient (ICC) was calculated to determine the agreement between measured and predicted RMRs. ICC results were interpreted as poor (below 0.5), moderate (0.50–0.75), good (0.75–0.90) and high (0.90 or higher) (Koo and Li, 2016). The root mean square error (RMSE) was calculated to indicate the model’s predictive performance in our data. A lower RMSE indicates a better performance of the RMR equations in estimating the actual RMR. The chi-square test was performed to compare the percentage of RMR prediction accuracy in participants grouped by gender and physical activity level. Statistical analyzes were performed using IBM SPSS Statistics, version 23.0 (IBM Corp., Armonk, New York) and R Studio using the “Bland Altman Leh” package (R Studio, Dusseldorf, Germany). The statistical significance level was accepted as *p* < 0.05.

## Results

Nine RMR measurements were repeated due to violations of RMR measurement requirements [non-compliance with pre-measurement rules; being a dehydrated state (*n* = 5), heavy exercise (*n* = 3) or smoking (*n* = 1) before measurement], indicating that 106 RMR measurement tests were completed.

Descriptive statistics of the participants are presented in Table 1. There were no significant differences between the groups in terms of age and BMI values (*p* = 0.067 and *p* = 0.109, respectively). Male and female athletes had significantly higher measured RMR and FFM (kg), and lower body fat percentage in those compared to the sedentary controls’ counterparts (*p* < 0.001).

**TABLE 1:** Descriptive statistics of the subjects.

	Males (*n* = 45)		Females (*n* = 48)	
	Athletes (*n* = 25)	Sedentary subjects (*n* = 20)	Athletes (*n* = 24)	Sedentary subjects (*n* = 28)
Age (year)	19.1 ± 1.5	19.9 ± 1.4	20.3 ± 2.1	20.1 ± 1.6
Body weight (kg)	75.4 ± 12.4	78.3 ± 12.8*	60.6 ± 12.7	60.0 ± 10.3*
Height (cm)	178.7 ± 6.1	176.8 ± 5.5*	163.3 ± 6.6	163.4 ± 6.1*
IC- RMR (kcal/day)	1,855.2 ± 322.4	1,366.0 ± 282.2*	1,366.0 ± 232.2	1,206.3 ± 161.7*
BMI (kg/m^2^)	22.9 ± 2.3	24.6 ± 3.0	22.7 ± 4.1	22.4 ± 2.9
Body fat (%)	10.6 ± 3.9	19.6 ± 4.1*	19.9 ± 4.5	26.7 ± 6.3*
FFM (kg)	66.7 ± 7.6	62.1 ± 5.7*	47.0 ± 5.7	43.4 ± 4.5*
TPAS (MET-min/week)	3,228.80 ± 426.22	534.11 ± 11.47*	3,009.13 ± 267.94	480.52 ± 34.15*

*p < 0.01. Values are expressed as mean ± SD. Independent samples t-test was performed to compare the means of descriptive characteristics between athletes and sedentary subjects, separated by gender.

*IC-RMR, Indirect Calorimetry-resting metabolic rate; BMI, body mass index; FFM, fat free mass; TPAS, total physical activity score; MET, metabolic equivalent.*

**FIGURE 1:**
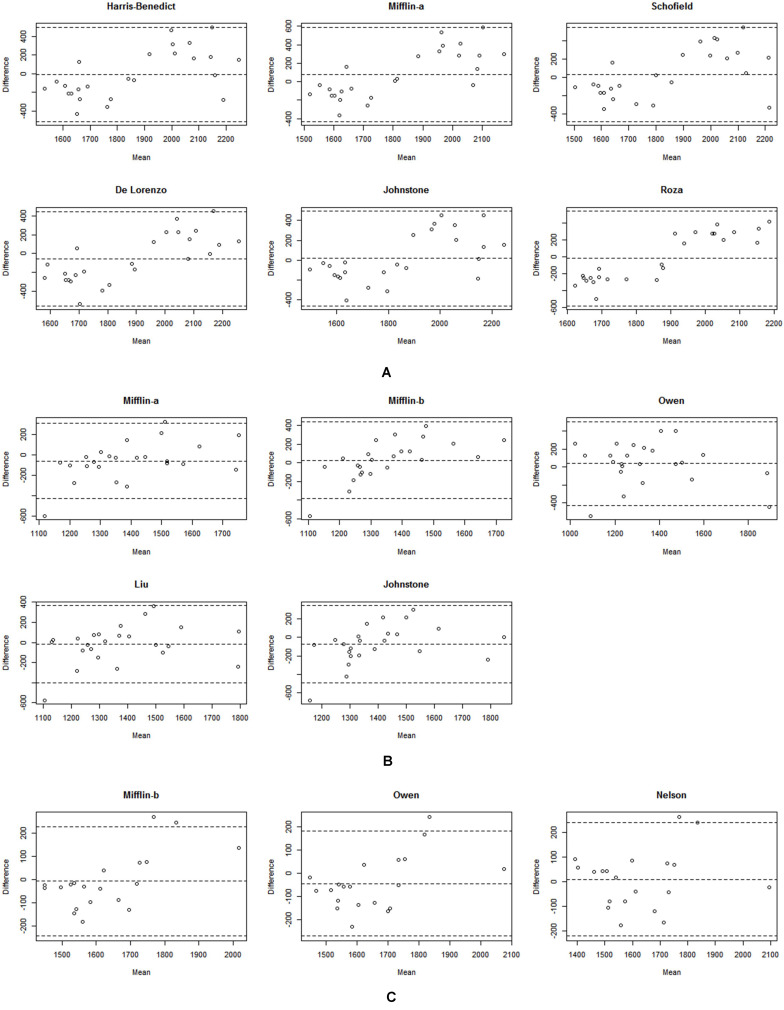
Bland–Altman plots for IC-RMR and predictive RMR equations for the subjects. The solid line represents the mean difference (BIAS) between RMR measured by Fitmate GS and predicted RMR. The upper and lower dashed lines represent the 95% limits of agreement. **(A)** Represents Bland-Altman plots of male athletes. **(B)** Shows Bland-Altman plots of female athletes. **(C)** Represents Bland-Altman plots of sedentary men. Mifflin-a indicates the formulation calculated using age, weight, and height. Mifflin-b indicates the formulation calculated using fat-free mass.

Table 2 summarizes the mean and mean differences between the measured RMR and the predictive RMR equations in the athletes. There were no significant differences between the measured RMR and Harris-Benedict, Mifflin (age, weight, height), Schofield, De Lorenzo, Johnstone, and Roza prediction equations in male athletes. The results of the Bland-Altman plot analysis for Harris-Benedict, Mifflin (age, weight, height), Schofield, De Lorenzo, Johnstone, and Roza equations in male athletes are presented in Figure 1A. A positive correlation value indicates that the predicted RMR is greater than the measured RMR. The relationship between average and bias of the measured RMR and predicted equations were significant for Harris-Benedict (*r* = 0.611), Mifflin (*r* = 0.711), Schofield (*r* = 0.703) De Lorenzo (*r* = 0.748), Johnstone (*r* = 0.609), and Roza (*r* = 0.903) equations in male athletes. The Bland–Altman plots for these predictive equations compared with the measured RMR showed proportional bias. The Harris-Benedict equation presented the most accurate RMR prediction in all RMR prediction equations with the lowest RMSE value of 252 kcal/day (Table 3). 40% of male athletes presented accurate values when using the Harris and Benedict equation; this percentage is statistically similar to the 50% observed in female athletes, but different than the 25% observed in sedentary men and 29% in sedentary women. However, ICC results showed that the Harris-Benedict equation (ICC = 0.524) had a moderate reliability observed in male athletes (Table 2).

**TABLE 2:** Comparison of IC-RMR and prediction equations in participants.

	Male Athletes (*n* = 25)			Female athletes (*n* = 24)			Males (*n* = 20)			Females (*n* = 28)		
	Mean ± *SD*	Mean difference	ICC	Mean ± *SD*	Mean difference	ICC	Mean ± *SD*	Mean difference	ICC	Mean ± *SD*	Mean difference	ICC
IC-RMR	1,855.2 ± 322.5			1,366.1 ± 232.2			1,628.1 ± 185.3			1,206.3 ± 161.7		
Harris-Benedict	1,864.1 ± 180.2	−8.9 ± 257.5	0.524	1,482.9 ± 142.5	−116.9 ± 191.7*	0.434	1,863.6 ± 188.9	−235.5 ± 122.7*	0.441	1,495.5 ± 186.8	−289.1 ± 164.9*	0.235
Mifflin^a^	1,777.8 ± 138.3	77.5 ± 260.8	0.436	1,425.2 ± 148.1	−59.1 ± 189.5	0.513	1,772.0 ± 145.4	−143.9 ± 113.9*	0.562	1,422.0 ± 186.7	−215.7 ± 159.9*	0.332
Mifflin^b^	1,726.9 ± 149.4	128.3 ± 241.9*	0.483	1,148.7 ± 111.4	27.1 ± 211.4	0.332	1,636.2 ± 112.2	−8.1 ± 120.5	0.700	1,268.5 ± 88.2	−62.2 ± 124.7*	0.494
Schofield	1,827.9 ± 186.4	27.4 ± 263.9	0.506	1,466.3 ± 197.8	−100.6 ± 205.9*	0.501	1,870.8 ± 193.3	−242.7 ± 123.9*	0.434	1,478.4 ± 226.2	−272.0 ± 196.9*	0.257
Cunningham	1,968.7 ± 166.9	−113.4 ± 237.8*	0.518	1,534.1 ± 124.4	−168.0 ± 212.5*	0.253	1,866.1 ± 125.4	−237.9 ± 117.3*	0.324	1,455.4 ± 98.5	−249.1 ± 123.7*	0.211
Owen	1,644.4 ± 126.3	210.8 ± 270.5*	0.290	1,324.7 ± 144.6	41.4 ± 214.6	0.386	1,673.4 ± 131.0	−45.3 ± 115.4	0.722	1,353.6 ± 192.4	−147.3 ± 185.8*	0.342
Liu’s	1,726.3 ± 179.6	128.9 ± 257.7*	0.465	1,382.8 ± 184.0	−16.7 ± 195.0	0.575	1,742.6 ± 188.0	−114.5 ± 120.1*	0.673	1,376.5 ± 194.7	−170.2 ± 162.4*	0.409
De Lorenzo	1,911.1 ± 146.5	−55.8 ± 256.0	0.476	1,596.7 ± 163.9	−230.6 ± 201.9*	0.303	1,914.5 ± 153.6	−286.4 ± 112.7*	0.325	1,593.1 ± 148.6	−386.8 ± 125.9*	0.164
Bernstein	1,510.9 ± 154.4	344.3 ± 244.0*	0.280	1,596.7 ± 127.9	217.4 ± 208.6*	0.231	1,447.3 ± 133.1	180.8 ± 114.5*	0.463	1,093.2 ± 104.0	113.1 ± 125.3*	0.432
Nelson	1,705.8 ± 200.6	149.5 ± 234.4*	0.543	1,339.0 ± 163.9	137.2 ± 216.5*	0.347	1,618.0 ± 170.1	10.1 ± 117.2	0.790	1,152.8 ± 133.0	53.6 ± 128.3*	0.594
Johnstone	1,837.8 ± 186.7	17.5 ± 242.7	0.584	1,228.9 ± 164.4	−72.7 ± 212.9	0.422	1,787.4 ± 173.5	−159.2 ± 116.2*	0.571	1,387.2 ± 136.1	−180.9 ± 132.0*	0.355
Roza	1,873.5 ± 85.3	−18.2 ± 284.7	0.279	1,483.2 ± 68.8	−255.0 ± 247.2*	−0.020	1,770.3 ± 27.3	−142.2 ± 117.3*	0.068	1,415.9 ± 51.3	−209.6 ± 143.1*	0.115

*p < 0.05. Values are expressed as mean ± SD. Paired samples t-test was performed to compare the means of measured resting RMR and RMR estimation equations, analyzed separately by sex and physical activity levels. ICC was calculated to determine the agreement between measured and predicted RMRs.

*IC-RMR, Indirect Calorimetry-resting metabolic rate; ICC, Intra-class correlation coefficient. ^*a*^Mifflin formulation calculated using age, weight and height; ^*b*^Mifflin formulation calculated using fat-free mass.*

**TABLE 3:** Accuracy, overprediction, and underprediction of each of the predictive equations compared to RMR measured by Fitmate GS in participants.

	Male athletes							Female athletes							Males							Females							
	A*		OP^+^		UP^++^			A*		OP^+^		UP^++^			A*		OP^+^		UP^++^			A*		OP^+^		UP^++^			
	n	%	n	%	n	%	RMSE	n	%	n	%	n	%	RMSE	n	%	n	%	n	%	RMSE	n	%	n	%	n	%	RMSE	p**
Harris-Benedict	10	40	9	36	6	24	252	12	50	10	42	2	8	221	5	25	15	75	0	0	264	8	29	20	71	0	0	331	**0.034**
Mifflin^a^	10	40	6	24	9	36	267	17	71	4	17	3	13	195	9	45	11	55	0	0	182	9	32	19	68	0	0	267	**0.037**
Mifflin^b^	15	60	2	8	8	32	419	14	58	4	17	6	25	298	16	80	2	10	2	10	212	13	46	13	46	2	7	167	0.218
Schofield	11	44	7	28	7	28	261	13	54	9	38	2	8	223	6	30	14	70	0	0	265	9	32	19	68	0	0	330	0.293
Cunningham	10	40	13	52	2	8	260	13	54	9	38	2	8	225	5	25	15	75	0	0	271	7	25	21	75	0	0	334	0.106
Owen	12	48	1	4	12	48	259	9	38	14	58	1	4	267	16	80	3	15	1	5	264	11	39	15	54	2	7	277	**0.025**
Liu’s	12	48	3	12	10	40	339	16	67	5	21	3	12	236	12	60	8	40	0	0	121	12	43	16	57	0	0	235	0.309
De Lorenzo	10	40	10	40	5	20	284	10	42	14	58	0	0	192	3	15	17	85	0	0	164	1	4	27	96	0	0	233	**0.002**
Bernstein	5	20	0	0	50	80	257	4	17	2	8	18	75	304	8	40	0	0	12	60	307	15	54	0	0	13	46	406	**0.001**
Nelson	15	60	2	8	8	32	270	8	33	2	8	14	58	209	16	80	2	10	2	10	118	16	57	2	7	10	36	137	**0.001**
Johnstone	13	52	6	24	6	24	274	13	54	7	29	4	17	252	11	55	9	45	0	0	115	10	36	18	64	0	0	137	0.457
Roza	6	24	11	44	8	32	310	9	38	15	63	0	0	232	8	40	11	55	1	5	133	7	25	21	75	0	0	294	0.517

**p < 0.05. Chi-squared test was used to compare the accurate predictions in participants grouped according to the gender and physical activity level.

RMSE was calculated to indicate the model’s predictive performance in our data.

*The percentage of participants predicted by the RMR equation within ± 10% of the RMR measured by Fitmate GS.

*^+^The percentage of participants predicted by the RMR equation within > 10% of the RMR measured by Fitmate GS. ^++^The percentage of participants predicted by the RMR equation within < 10% of the RMR measured by Fitmate GS.*

*A, Accurate; OP, Over-Predicted; UP, Under-Predicted; RMSE, root-mean-squared prediction error.*

Mifflin (age, weight, height), Mifflin (FFM), Owen, Liu’s, and Johnstone predictions did not significantly differ compared to the measured RMR in female athletes (Table 2). Figure 1B shows the Bland-Altman plot results for the Mifflin (age, weight, height), Mifflin (FFM), Owen, Liu’s, and Johnstone prediction equations in female athletes. The relationship between the average and the bias of the measured RMR and the predicted equations was significant for Mifflin (age, weight, height) (*r* = 0.422), and Mifflin (FFM) (*r* = 0.662) predictions but was not significant for the Liu’s (*r* = 0.277), Owen (*r* = 0.182), and Johnstone (*r* = 0.370) predictions in female athletes.

With the highest reliability seen in Liu’s equation (ICC = 0.575), Mifflin (age, weight, height) and Liu’s equations had a moderate reliability in female athletes. In addition, Mifflin (FFM), Owen and Johnstone equations showed a low reliability. The Liu’s equation presented an accurate RMR prediction with the RMSE value of 236 kcal/day, predicting RMR of 67% of the subjects (Table 3). This percentage is also statistically similar to the 48% observed in male athletes, 60% in sedentary men and 43% in sedentary women. However, the ICC results showed that the Liu’s equation (ICC = 0.575) had a moderate reliability observed in female athletes (Table 2).

Table 2 presents the mean differences between the measured and the predicted RMRs in the sedentary subjects. The ICC results indicate that Mifflin and Owen equations were moderately reliable, with the highest reliability observed in the Nelson equation in sedentary men (ICC = 0.790) (Table 2). Mifflin (FFM), Owen and Nelson equations predict RMR of 80% of the subjects by presenting 118–212 kcal/day RMSE values in sedentary men (Table 3). This percentage of accurate predictions is statistically similar to the 60% observed in male athletes, but not similar to the 33% observed in female athletes and 57% observed in sedentary women. The Bland-Altman plot revealed that the relationship between average and bias of the measured RMR was significant for the Mifflin (FFM) (*r* = 0.641), and the Owen equations (*r* = 0.497) but was not significant for the Nelson (*r* = 0.137) equation in sedentary men (Figure 1C).

In sedentary women, there was a significant difference between the measured RMR and all equations (*p* < 0.001) (Table 2).

## Discussion

The main objectives of the study were to estimate the RMR using the Harris-Benedict, Mifflin (age, weight, height), Mifflin (FFM), Schofield, Cunningham, Owen, Liu’s, De Lorenzo, Bernstein, Nelson, Johnstone, and Roza equations, and to compare these results with the RMR measured in Turkish Olympic young adult national team athletes and their sedentary counterparts and determine the suitability of these equations for participants. The main findings rejected our hypothesis, revealing that the predictive RMR equations have a low to moderate absolute agreement with the measured RMR at the individual level due to a wide range of limits of agreement. Therefore, these equations did not accurately predict RMR in young adult Olympic national team athletes and their counterparts.

This is the first study to evaluate the RMR of Olympic young adult national team athletes and their counterparts by comparing estimated RMR equations with those measured by Fitmate GS. According to the gender of the participants, the measured RMR was higher in athletes than in their sedentary counterparts. Additionally, the FFM results showed that the athletes had a higher FFM compared to the sedentary controls. It is well-known that FFM is one of the major determinants of RMR (Blundell et al., 2012). Several studies have documented that FFM shows a greater correlation with RMR compared to fat mass, age, and BMI (Johnstone et al., 2005; Blundell et al., 2012). This correlation was interpreted as the fact that muscles were more metabolically active compared to adipose tissue (Leskinen et al., 2010). Along with the data, several RMR predictive equations have been developed based on FFM (Cunningham, 1991; Nelson et al., 1992). Therefore, we may suggest that the higher RMR in athletes may be due to athletes having higher FFM values than their sedentary counterparts. The findings also emphasized that RMR prediction equations validated on sedentary subjects would not be valid for predicting RMR in athletes. It may be best to first determine the accuracy and validity of the RMR estimation equations before applying them to athletes in practice.

A well-designed diet combined with an effective training program is the core component of athletic preparation and, if done correctly, can determine their success in Olympic sports (Close et al., 2016). In a situation where adequate energy intake is not provided, various nutrient deficiencies and injuries can occur, and as a result, may negatively impact sport performance and lead to poorer health outcomes (Mountjoy et al., 2014, 2018). Therefore, an accurate RMR estimation becomes crucial when RMR cannot be measured with indirect calorimetry.

The Harris-Benedict equation seemed to be the closest estimate for male athletes, with the lowest RMSE value of 252 kcal/day accurately estimated the RMR of 40% of the participants. This estimate is also statistically similar to the 50% observed in female athletes. However, the ICC results showed a moderate relative agreement (0.524). Further, the Bland-Altman plots of male athletes indicated that there was a significant (*p* < 0.001) proportional bias in Harris-Benedict (*r* = 0.611), and also in Johnstone (*r* = 0.609), Mifflin (*r* = 0.711), Schofield (*r* = 0.703), De Lorenzo (*r* = 0.748), and Roza (*r* = 0.903) equations. These findings suggest that the predictive RMR equations underestimate the high RMR values, and overestimate the low RMR values. For female athletes, although Liu’s equation was found to be the most accurate equation predicting accurately in 67% of the subjects (RMSE = 236 kcal/day) and no proportional bias, ICC results showed moderate relative agreements between the measured RMR and Liu’s equation (ICC = 0.575). It appears that 236 kcal/day may be a small overprediction, but it affects the total energy expenditure by 401.2–566.4 kcal/day as physical activity level calculated by RMR^∗^physical activity level (PAL). The PAL coefficient ranges from 1.7 to 2.4 depending on moderately active to severely active lifestyle (Joint FAO/WHO/UNU Expert Consultation, 1985). Additionally, it is well defined that the PAL coefficient differs depending on the intensity and duration of the training even among athletes doing the same sport. For example, average PAL was found to be 1.71 for collegiate swimmers with intermediate training, while for elite swimmers, PAL was found to be 3.0 for men during higher training (Park, 2019). This underestimation of energy needs can create a huge energy deficit in this athlete’s diet prescription, resulting in loss of body weight and relative energy deficiency syndrome with various critical symptoms including metabolic, physiological, immunological issues (Mountjoy et al., 2014, 2018). Conversely, the overestimation of energy requirements can result in a weight gain that will attenuate the performance of a Olympic young adult athlete (Thomas et al., 2016). Considering all this data, RMR prediction equations significantly misestimate the RMR in Olympic young adult national team athletes.

In the latest ACSM position stands, the Harris-Benedict and Cunningham equations were recommended for estimating RMR in the athletic population (Thomas et al., 2016), which is inconsistent with our study results. However, it remains equivocal whether these equations are applicable to all athletic populations. Few studies have evaluated the interaction between measured vs. predicted RMR in athletes with various sports disciplines (Thompson and Manore, 1996; Carlsohn et al., 2011; Juzwiak et al., 2016; Jagim et al., 2018; Devrim-Lanpir et al., 2019; Schofield et al., 2019). Accordingly, these studies had suggested that certain equations are more suited to specific athletic populations. For example, the Owen and Mifflin equations for Paralympic track and field athletes (Juzwiak et al., 2016), the Mifflin and Cunningham equations for male ultra-endurance athletes (Devrim-Lanpir et al., 2019), the Mifflin equation for female ultra-endurance athletes (Devrim-Lanpir et al., 2019), the Harris-Benedict equation for NCAA Division III male athletes (Jagim et al., 2018), and the Cunningham equations for NCAA Division III female athletes (Jagim et al., 2018). Others studies have declared that the current predictive RMR equations underestimate RMR in athletes such as bodybuilders (Joseph et al., 2017), adolescent athletes (Reale et al., 2020), and heavyweight endurance athletes (Carlsohn et al., 2011), and as a result may not be suitable for the use in these populations. For Olympic young adult national team athletes, our findings revealed that none of the RMR prediction equations used in the study predicted RMR accurately. Therefore, a new equation is needed to estimate RMR. The main reasons for the different results may be due to the difference in the study design, the IC metabolic device, the measurement protocol and the athletic population. For the measurement protocol, there is no consensus on measuring RMR using the IC. This causes differences in the measurement protocol between studies. For example, studies applied different rest periods before measuring RMR (Fullmer et al., 2015; Yeung et al., 2020), while Compher et al. (2006) recommend resting for at least 20 min before the measurement. Therefore, a standardized measurement protocol is required to measure RMR in order to accurately compare study results.

One possible explanation why the predictive RMR equations do not estimate RMR accurately is that the predictive RMR equations are often developed based on data from different populations. In these studies, the investigated populations included healthy adults (Harris and Benedict, 1918), obese adults (Mifflin et al., 1990), trained adults (Cunningham, 1991) and athletes (De Lorenzo et al., 1999), and persons from various ethnic backgrounds (Harris and Benedict, 1918; Liu et al., 1995; ten Haaf and Weijs, 2014). Therefore, any differences between the participants may be a result of the applied and specifically validated RMR equation, which may be influenced by several other factors, including overall metabolic health status, ethnicity, athletic training history, and developmental age.

Five of the equations we used to predict RMR, including Cunningham, Mifflin, Bernstein, Johnstone, and Nelson, were FFM-based equations. However, these equations were found not to predict RMR accurately. One of the possible reasons is that FFM-based equations were generally validated in non-athletic populations such as normal weight (Mifflin et al., 1990; Nelson et al., 1992; Johnstone et al., 2006), and obese adults (Bernstein et al., 1983; Mifflin et al., 1990; Nelson et al., 1992; Johnstone et al., 2006). Therefore, these RMR prediction equation may underestimate the actual RMR results due to the reason that FFM is greater in athletes compared to non-athletic populations.

One of the strengths of this study was the inclusion of age- and BMI-matched sedentary controls. This allowed for a more in-depth comparative analysis of the metabolic and physiological characteristics of national and Olympic level athletes with matched controls. Thus, we were able to emphasize that RMR prediction equations validated in sedentary participants may not be accurate and valid for the athletic population. Another strength of our study is to use of a ventilated canopy hood instead of a face mask. This provided a more comfortable measurement and eliminated the possibility of air leakage from the system. Face masks, even with many different sizes, sometimes may not fit all faces properly and may cause gas leakage during testing, even with great care before the measurements. In addition, although individuals are allowed to get used to the mask before the test, it may be disturbing during the measurement process. Since we used only a ventilated canopy hood, we had now chance to compare these two equipments. However, all participants reported that they felt comfortable during the measurement.

In this study, RMR measurements were performed using the Fitmate GS. The measurement of RMR would be more accurate if we would have used other more advanced systems measuring both oxygen consumption and carbon dioxide production. However, the Fitmate GS metabolic cart is validated against the “gold standard” Douglas bag (Nieman et al., 2006) and Quark CPET (Vandarakis et al., 2013), a previously validated metabolic device, in healthy adults and reveals little error in estimating RMR as confirmed in previous studies (Nieman et al., 2006, 2007). Additionally, we carefully ran its validation and calibration-related tests before and during the measurement period and controlled all variables (e.g., room temperature, noise, pre-check, and calibration of the analyzer, food/beverage/nicotine/alcohol consumption, exercise) that could affect the accuracy of the RMR results.

One limitation of the study is that we had no control group matched by FFM. Due to the differences in training status, we had serious difficulties to find subjects for an FFM-matched control group. However, this study emphasized that the FFM values of athletes were significantly different from their sedentary counterparts. Therefore, more validated RMR estimation equations are needed in athletic populations, particularly in athletic young adults.

We applied the IPAQ-short form to determine the physical activity level of the participants. Since there is no gold standard criterion for measuring physical activity level (Terwee et al., 2010) and motion sensors, such as triaxial accelerometers, are practically not feasible due to their high cost and time-consuming features (Lee and Shiroma, 2014), it is widely preferred to determine the level of physical activity by applying a questionnaire. Although the interaction between the IPAQ-SF and objective measures of physical activity in several studies was lower than the acceptable standard (Lee et al., 2011), it was found reliable and valid in determining total physical activity in Turkish participants aged 18–32 years (Saglam et al., 2010). Considering that the individuals participating in our study were included in this age group, the use of the IPAQ-short form is considered as an appropriate and valid method.

We used a multi-frequency bioelectrical impedance analyzer to determine body composition. Although bioelectrical impedance analysis is not a gold standard method to determine body composition assessment, it is a validated, easy-to-use method developed as an alternative to more expensive and invasive gold standard methods such as dual-energy X-ray absorptiometry (DXA) and magnetic resonance imaging to estimate body composition. As stated by Verney et al. (2015), BIA results provide satisfactory measurements for fat mass and fat-free mass compared to DXA measurement results in healthy young adults regardless of their physical activity levels. However, in order for a valid and accurate test, it is crucial to ensure the necessary conditions before the test is performed (Kyle et al., 2004). For this reason, before all BIA measurements, we checked that all the necessary conditions for accurate measurement were met (e.g., food/drink restriction, hydration status, analyzer calibration, and room temperature) (Kyle et al., 2004).

Additionally, sleep loss could be another factor to alter RMR results (De Jonge et al., 2012). Since we did not determine sleep loss or quality, we cannot define if it had an effect on the RMR measurement. The impact of sleep quality should be investigated in future studies.

Due to the small number of participants and the inclusion of athletes from different sports disciplines, we could not perform a regression analysis. Therefore, we could not develop group-specific predictive RMR equations. However, our current findings highlight the urgent need for future studies on a new predictive RMR equation to accurately measure the RMR of Olympic young adult athletes.

Our study’s findings demonstrate a low to moderate relative agreement between the measured RMR and the predictive RMR equations. We know that RMR is one of the major components of total energy needs. Although some other studies applied in athlete populations (Thompson and Manore, 1996; Carlsohn et al., 2011; Juzwiak et al., 2016; Jagim et al., 2018; Devrim-Lanpir et al., 2019; Schofield et al., 2019), according to our knowledge, none of them collected data from Olympic young adults. Although any of these RMR prediction equations have not been validated on the Olympic young adults, they are widely used in calculating energy needs of athletes due to the lack of indirect calorimetry in all Olympic centers. Therefore, we sought to investigate the interactions between widely used RMR equations and the IC RMR measurement to determine the accuracy of these RMR predictions and detect the best accurate RMR prediction equation for Olympic young adults. Therefore, considering the importance of the appropriate determination of energy expenditure, it may not be suitable to use these equations as a component in calculating the total energy needs of Olympic young adult national team athletes. If possible, it is recommended that RMR in Olympic young adult athletes should be measured by using an IC. Otherwise, further studies should be applied in a larger cohort of Olympic young adult national team athletes to develop a group specific RMR prediction equation.

## Data Availability Statement

The raw data supporting the conclusions of this article will be made available by the authors, without undue reservation.

## Ethics Statement

All procedures of the study were conducted in accordance with the Declaration of Helsinki. The ethics committee approval was obtained from the University Ethics Committee (05.02.2020/37). The patients/participants provided their written informed consent to participate in this study.

## Author Contributions

AB, EB, AY, BA, TK, and AH contributed to the study design and conception. EB, BA, TK, and AH completed the data acquisition. AY completed the data analysis. AB, AD-L, EB, and BK completed the writing-original draft preparation. AB, EB, AY, AD-L, BA, TK, AH, LH, TR, and BK completed the review of the final manuscript. All authors contributed to the article and approved the submitted version.

## Conflict of Interest

The authors declare that the research was conducted in the absence of any commercial or financial relationships that could be construed as a potential conflict of interest.
